# OLDER LGBTQ+ PEOPLE’S PERSPECTIVES ON DISCRIMINATION AND AGEING IN THE UK: EMERGING FINDINGS FROM A TIMELINE STUDY

**DOI:** 10.1093/geroni/igad104.3758

**Published:** 2023-12-21

**Authors:** Michael Thomas, Amy Prescott, Christina Victor

**Affiliations:** Brunel University London, Uxbridge, England, United Kingdom; Brunel University London, Uxbridge, England, United Kingdom; Brunel University London, Uxbridge, England, United Kingdom

## Abstract

This paper presents emerging findings from a United Kingdom study of socially inclusive ageing, reporting on timeline interviews with a sample of 25 LGBTQ+ people aged forty years and above, carried out during summer and fall of 2023. The research interrogates older LGBTQ+ people’s understanding of the impact of minority sexuality and gender identity on their lived experience and the ways in which this impacts their approach to challenges and opportunities linked to ageing. We draw on an innovative methodological approach, adapting Adriansen’s (2012) timeline interview method to fit the needs of older participants. Timeline interviews provide an approach to life-story research that structures a conversation around a written timeline, produced on paper during unstructured qualitative interviews. During the paper presentation we share anonymised timeline and narrative data highlighting milestones in individual biographies. We focus on moments and periods where sexuality and gender identity may have been a positive factor in individual life stories (romantic relationships, community membership) or have been seen as more problematic (discrimination, stigma and marginalisation). The timelines and associated interview data also provide opportunities to ground the biographies of older LGBTQ+ people in the context of social, cultural, political and policy contexts. The timelines reveal compelling data about ageing and identity, belonging and exclusion. Our emerging findings reflect C. Wright Mills’s concept of the sociological imagination, placing personal troubles and histories in the context of public issues. We gratefully acknowledge the funding this study has received from the United Kingdom Economic and Social Research Council.

